# Endangered Galápagos sea lions and fur seals under the siege of lethal avian flu: a cautionary note on emerging infectious viruses in endemic pinnipeds of the Galápagos Islands

**DOI:** 10.3389/fvets.2024.1457035

**Published:** 2024-09-20

**Authors:** Juan José Alava, Ana Tirapé, Judith Denkinger, Paola Calle, Patricia Rosero R., Sandie Salazar, Patricia A. Fair, Stephen Raverty

**Affiliations:** ^1^Ocean Pollution Research Unit, Institute for the Oceans and Fisheries, University of British Columbia, Vancouver, BC, Canada; ^2^School of Resource and Environmental Management, Simon Fraser University, Burnaby, BC, Canada; ^3^Fundación Ecuatoriana para El Estudio de Mamíferos Marinos (FEMM), Guayaquil, Ecuador; ^4^Escuela Superior Politécnica del Litoral, ESPOL, Facultad de Ciencias de la Vida, ESPOL Polytechnic University, Guayaquil, Ecuador; ^5^Universidad San Francisco de Quito, Galápagos Science Center, Quito, Ecuador; ^6^Ocean Museum, Stralsund, Germany; ^7^Escuela de Ciencias Ambientales, Universidad Espíritu Santo, Samborondón, Ecuador; ^8^Department of Public Health Sciences, Medical University of South Carolina, Charleston, SC, United States; ^9^Animal Health Centre, British Columbia Ministry of Agriculture, Food and Fisheries, Abbotsford, BC, Canada

**Keywords:** Galapagos sea lion, Galápagos fur seal, avian A(H5N1) influenza, emerging infectious diseases (EIDs), mortality, emergency preparedness (EP), epizootic, Galapagos marine reserve

## Introduction

The avian influenza HPAI H5N1 panzootic emerged in wild birds and poultry from the Northern Hemisphere in 2020 and has disseminated globally (1–6). From 2016 to 2023, the H5N1 clade 2.3.4.4b was confirmed in mustelids (Mustelidae), pinnipeds (Phocidae), and cetaceans (Delphinidae and Phocoenidae) in more than 24 European countries (7–9). Additional cases of H5N1virus infections in marine mammalian species (e.g., otariids, phocids, and delphinids) have subsequently been reported in Europe, North America, South America, West and South Africa (8, 9), while few reports of H5N1 virus prevalence have been documented in Australasia, with the detection of the H5N1 clade 2.3.4.4b in Bangladesh but a lack of detection of this clade in Australia (10, 11). Then, between October and November 2022 and in 2023, the H5N1virus was confirmed along South American coasts, including Argentina, Chile, Perú, Brazil, Uruguay, the Antarctic circumpolar regions (2, 3, 5, 7, 12–16), and two remote biological diversity hotspots, the UNESCO World Heritage site Galápagos Islands (Ecuador) in September 2023, and South Georgia (sub-Antarctic) in December 2023 (7, 17). Depending on the species of wildlife, cumulative mortalities in South America due to highly pathogenic H5N1 have ranged from 3,000 to 7,000 to over 260,000 animals, including thousands of seabirds and pinnipeds that have succumbed to this deadly virus (14, 18–20). A timeline of the HPAI (H5N1) events affecting marine mammals in South America in chronological order with references from February 2021 to July 2024 is documented in Supplementary Table 1.

Although H5N1 virus variant infections have not previously been found in the past within the Galapagos Islands (17), massive commercial poultry losses in Ecuador have been attributed to the highly pathogenic influenza A H5N1 2.3.4.4b clade (21). This virus variant is suspected as the cause of the mortality of frigatebirds (*Fregata* sp.) and several red-footed boobies (*Sula sula*) on Genovesa and Wolf islands and Punta Pitt at San Cristóbal Island (17). Subsequent laboratory analysis confirmed that red-footed boobies and frigate birds and other free-ranging avian species, including the Galapagos red-billed tropical bird (*Phaethon aethereus*), Nazca booby (*Sula granti*), and blue-footed booby (*Sula nebouxii*), were infected with the H5N1virus, likely exposed to the 2.3.4.4b clade. This virus was also detected in dead or dying seabirds (e.g., frigatebirds, blue-footed bobbies) from Ecuador's mainland coast and mangroves in November 2023. According to the Global Initiative on Sharing All Influenza Data (GISIAD), the influenza genome sequence subtypes detected in early 2024 in birds of the Galápagos and Ecuador's continental coast were the A/H1N1, A/H3N2, B/Victoria, and B/Yamagata (22). Nonetheless, the long-term population health impacts of the avian flu epizootic in endemic seabird colonies and potential horizontal transmission to endemic and endangered Galápagos sea lions (*Zalophus wollebaeki*) and Galápagos fur seals (*Arctocephalus galapagoensis*) across several islands have not yet been resolved. This situation is criticalsince the virus has the capacity to rapidly mutate, evolve, and infect other warm-blooded species such as marine mammals, and is of great concern to the conservation and management of these enigmatic species (19). Herein, we provide an overview on the detection of H5N1 avian influenza in seabirds of the Galápagos Islands and reports in mammals from other regions of the world (8, 18, 19) highlighting the potential epidemiological risks and health implications to endemic pinniped populations.

## The emerging H5N1 avian influenza in the Galápagos Islands

In contrast to prior outbreaks of highly pathogenic avian influenza, there are distinct differences in the spatiotemporal infections of the European H5N1 subtype, clade 2.3.4.4b in wild and commercial birds, as well as free-ranging wildlife (8, 23). Historically, in the European Union, there was a distinct seasonality with the number of HPAI detections in birds; however, with the current panzootic, infections have persisted from 2021 to early 2023 and the number and types of affected species have expanded (8). Aggregation in large numbers of susceptible animals is particularly prone to rapid viral dissemination once introduced. In South America, there has been a spillover of H5N1clade 2.3.4.4b to marine mammals with devastating impacts, which likely reflects a complex and dynamic transmission pathway (15), with discontinuous dissemination of the virus to susceptible host populations likely via migratory flyways. The recent detection of avian influenza in subantarctic and Antarctic seabirds reflects the further southward expansion of the virus range (24). Based on the natural history of the virus and the detection of other influenza virus types in the Galápagos Islands, H5N1 clade 2.3.4.4b poses a significant threat to endemic and native wildlife species.

While there is no historical evidence of prior exposure of Galápagos sea lions or fur seals to influenza viruses, serological surveys between 2001 and 2002 in Santa Cruz Island documented the local exposure in introduced mammals (i.e., dogs and cats) to other viral pathogens, including canine distemper virus (CDV; 50% or 7/14), canine parvovirus (14% or 1/7), and canine adenovirus (canine infectious hepatitis virus, 100% or 1/1) in the free-ranging, feral and companion canine population (25, 26). Similarly, seroepidemiological surveys and DNA sequencing conducted at Isabela Island detected exposure to and infection with several pathogenic viruses in dogs, including canine parvovirus, canine parainfluenza virus, canine adenovirus, and CDV, as well as feline panleukopenia virus, feline calicivirus, and feline herpesvirus 1 in cats (27). Moreover, as evidence of horizontal virus transmission from domestic dogs, Galapagos sea lion pups were already exposed to CDV over the past decade (28). One example occurred in the Galápagos sea lion colonies at Wreck Bay on San Cristóbal Island, where the death rate of juvenile and adult sea lions related to disease increased from 2008 to 2015, while pup mortality peaked in 2011 and remained high in 2012 (28). Most of the pups showed signs of upper respiratory tract infections; and, tissue samples collected in Wreck Bay during 2011–2012 confirmed the detection of CDV-RNA in six out of 48 Galápagos sea lion pups found dead in haul-out sites of Wreck Bay, where sea lions inhabit the harbor and urban environment of Puerto Baquerizo Moreno (28). Four of six pups (66.7%) were positive for CDV by nucleotide sequencing (28). Also, CDV was recently detected in a population of 125 dogs sampled at Santa Cruz, where the positivity rate detected via RT-qPCR reached 74.4% (29).

Through horizontal dissemination, these pathogenic viruses have posed a significant risk to fragile local endemic pinniped populations over the last two decades (26, 30–32). Recent studies suggested that H5N1 avian influenza viruses have increased pathogenicity in laboratory mice, indicating that the virus is becoming more virulent for terrestrial mammals (33). Thus, a looming threat exists for not only local CVD outbreaks from domestic dogs but also the ongoing regional epidemic and circulation of the H5N1 strain among South American sea lions and fur seals from colonies in Perú and Chile (19), as well as in coastal seabirds in the Galápagos Islands (17). Based on the precedent of non-influenza virus transmission and infection from companion animals, the risk of exposure to sea lions is a paramount concern for the population health, conservation, and survival of these endangered Galápagos pinnipeds.

Due to the propensity of Galápagos sea lions and fur seals to haul out in large numbers in rockeries and lack of prior exposure and development of protective immunity to H5N1, there is a strong likelihood of rapid viral dissemination. Based on reported mortalities in other marine mammal species along the Pacific coast of South America, the impact of infection on Galápagos sea lion and fur seal rookeries may be catastrophic. Colonies of Galápagos penguins (*Spheniscus mendiculus*), flightless cormorants (*Phalacrocorax harrisi*), and Galápagos albatrosses (*Phoebastria irrorata*) can also be exposed in the short-term. Along the Peruvian and Chilean coastlines, multiple large-scale mortality events have been reported for Peruvian pelicans (*Pelecanus thagus*), Guanay cormorants (*Leucocarbo bougainvillii*), and a close relative of the Galápagos penguin, the Humboldt penguins (*Spheniscus humboldti*), South American sea lions (*Otaria flavescens*), and several small-toothed cetacean species (e.g., common dolphin, *Delphinus delphis*; Chilean dolphins *Cephalorhynchus eutropia*; Burmeister's porpoise*, Phocoena spinipinnis*), with sporadic cases in marine otters (*Lontra felina*) and the southern river otter (*Lontra provocax*) (19, 34–36). These events mark the first H5N1 spillover and avian flu mass mortality for these species in South America. In October and November 2023, the H5N1 clade 2.3.4.4b caused the devastating mass mortality of >17,000 southern elephant seal pups (*Mirounga leonina*), accounting for over 95% of the entire population in a single continental colony on Península Valdés, Argentina (18). These mass die-off events observed along the South American coasts raise serious concerns for other pinniped populations, particularly threatened or endangered species with small, geographically confined breeding populations such as Galápagos sea lions and fur seals, in the southeastern tropical Pacific.

**Figure 1:**
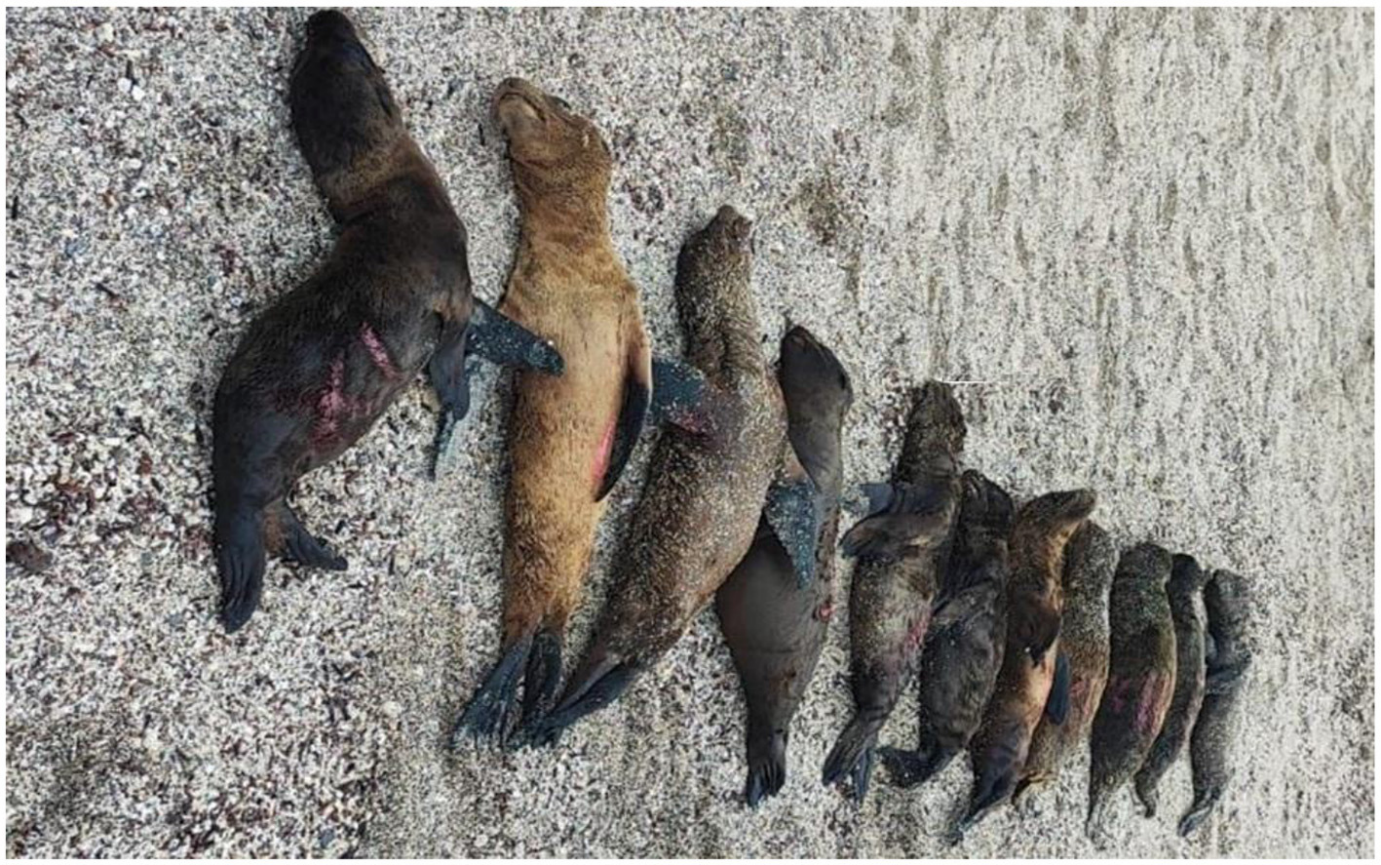
Photo showing 11 Galápagos sea lion (*Zalophus wollebaeki*) pups of similar age and size found deceased at the La Lobería rookery in San Cristóbal Island (Galapagos Islands, Ecuador) on 9 January 2024. Lateral, red lesions marked with red paint for individual identification purposes can be observed on the ventral and dorsal regions of the pups. Photo credit: BitacoraAmbiental Ecuador @BitacoraEc (9 January 2024), available at: https://x.com/BitacoraEc/status/1744822075582288162?s=20.

In early January 2024, 11 Galápagos sea lion pups were found dead on a rookery (La Lobería), located at San Cristóbal Islands (Figure 1). According to the field observations and inspections of dead pups, the Galápagos National Park and Ecuador's Ministry of Environment, Water and Ecological Transition reported the presence of wounds on the pups, concluding that the lesions were associated with bites inflicted by feral or stray dogs. However, given the external appearance, size and shape of apparent lesions in the dead pups (Figure 1), this mass mortality event was unlikely to be associated to dog attacks alone. No injuries were seen in other sea lions at the same rookery the next day (J. Denkinger, personal observation, 10 January 2024, USFQ, Galápagos Science Center, San Cristóbal, Galápagos, Ecuador), which would have been a typical scenario for a case where feral dogs attack a group of about 30 sea lions. Thus, questions remain on the association of the pups' mortality to a dog attack. No further clinical or diagnostic information on the cause of death or any pre-existing pathology was reported by the authorities. Without a conclusive cause of death for the pups, the possibility that these pups from La Lobería rookery died to an infection such as CDV (28), malnutrition (i.e., nutritional stress linked to more frequent, intense El Niño events or El Niño Southern Oscillation-ENSO (30, 32, 37, 38), maternal neglect and sibling conflict (39), attempted predation (40), as well as other diseases processes and environmental factors or anthropogenic stressors, cannot be ignored (28, 31, 32, 37, 41).

## Discussion

Small populations of endemic and endangered Galápagos pinnipeds and seabirds are currently affected by multiple anthropogenic stressors, including marine pollution, fishing pressure, and climate change (31, 32, 41, 42). These multiple anthropogenic stressors are further exacerbated by periodic El Niño and ocean warming (ENSO episodes) events, which may cumulatively impact small teetering populations of endemic species in the Galápagos (32, 38, 42). A La Niña event cold conditions was anticipated in mid-2024 and may further influence viral dissemination among the susceptible endemic seabirds and pinnipeds in the Galápagos. These environmental processes may predispose and possibly exacerbate host susceptibility to emerging pathogens (31, 32, 41). In the Galapagos Islands, epidemiological monitoring and surveillance efforts to detect and eradicate avian flu are led by the Control and Regulation of Biosecurity and Quarantine Agency in collaboration with the Galápagos National Park Directorate. These local agencies have enacted rigorous biosecurity and sanitary protocols with restricted ecotourism to visit islands harboring seabirds exposed to the influenza virus.

In addition to the regional emergency response measures in the Galápagos and Ecuador's continental coast to limit the dissemination of the avian flu across the islands, we emphasize the development of appropriate reporting strategies, sampling protocols, case definitions, use of personal protection equipment (PPE), and epidemiological data sharing, as outlined in Table 1. Table 1 illustrates a high-level strategic direction to support and inform the development and/or revision of the national and regional action plans for HPAI H5N1 prevention and control intended to be adapted for the animal health sector including the wildlife component (with a particular focus on wildlife where animal health interventions can significantly reduce HPAI H5N1) and public health of a wider One Health approach to the prevention and control of HPAI H5N1 (43).

**Table 1:** Proposed strategic actions and recommendations to reinforce the Galápagos Islands preparedness and surveillance plan to combat HPAI H5N1 influenza virus epizootic in Ecuador in close conjunction with the Control and Regulation of Biosecurity and Quarantine Agency and the Galápagos National Park Directorate.

**Pillars for strategic actions**	**Recommendations**
(1) Galápagos Islands' emergency preparedness for coordination, planning, and monitoring	•Developing and implementing an official Galápagos and National Action Response and Preparedness Plan for the Contingency of HPAI H5N1 to strengthen current efforts by the Control and Regulation of Biosecurity and Quarantine Agency: - Isolate infected native and endemic species (e.g., seabirds), domestic animals and people and ensure infection control - Closure of regional/national borders - Quarantine for arriving tourist and travelers - Intense testing and confirmation of animal (e.g., endemic species such as seabirds and shorebirds) and human cases - Implement contact-tracing capabilities to track all affected wildlife, and domestic animals as well as contact people exposed to the virus via dead or moribund animals (wildbirds, including land birds, waterfowl, shorebirds and seabirds, as well as poultry farms) or humans that were in contact with an infected person and quarantine all of them - Use of PPE (masks) and social distancing - Monitor as to whether a decreasing number of cases is occurring over a given period of time (i.e. every 15 days weeks or after a month)
(2) Risk communication and community engagement	•Promoting and diffusing public messaging and health education programs addressed to the population (e.g., prevent and avoid contact with dead seabirds, marine mammals, domestic and feral mammals (e.g., dogs, cats,); practicing social distancing at 2–4 m and home isolation, promoting PPE or mask use)
(3) Surveillance, rapid response teams, and case investigation	•Ensuring that a local/regional Emergency Response Animal Health Team was already assembled •Concerted monitoring of new H5N1 cases and hospitalized wildlife and domestic animals and patients and recovered species and people. •Rapid animal and public testing and implement contact tracing
(4) Points of entry	•Informing travelers of prevention measures •Establishing a tentative quarantine period for foreign citizens, visitors, tourists, international and residents arriving to Ecuador and the Galápagos Islands from overseas. •Setting up temporal closure of the Galápagos Island's territory borders
(5) Regional and National laboratories,	•Adopting standardized systems for molecular testing, supported by assured access to reagents and kits •Sharing genetic sequence data and virus materials according to established protocols for HPAI H5N1 virus
(6) Infection prevention and control,	•Strengthen the availability of proper PPE for veterinarians, health workers, and field research personnel (e.g., veterinary doctors, wildlife epidemiologists Galápagos National Park rangers, wildlife biologists and field assistants) •Secure keeping animal and health care centers and facilities safe as top priority •Ensure aggressive containment of virus as top priority and intense infection control
(7) Case management	•Mapping vulnerable wildlife and animal populations and public and private health facilities and identify alternative facilities that may be used to provide treatment •Identifying Intensive clinical and care unit capacity for infected animals and people •Ensuring comprehensive medical, nutritional, and care for animal species and those with H5N1
(8) Operational support and logistics	•Assessing the capacity of the local market to meet increased demand for medical and other essential supplies, and coordinate international requests for supplies through regional and global procurement mechanisms (e.g., World Organization for Animal Health and World Health Organization) •Identifying potential major impacts on the transportation systems of health supplies, education, and food transportation networks

Based on and adapted from Alava and Guevara (45).

Developing an effective multidisciplinary monitoring and surveillance program to combat viral disease affecting threatened marine-coastal fauna, including marine mammals and sea birds, from remote oceanic islands is imperative. Emergency preparedness, early detection, immediate notification, and biosecurity response, including vaccines against the avian flu (4), are fundamental in preventing strategies to protect biodiversity and humans (23, 44). In this context, it is important to implement strict biosecurity and sample collection protocols for *in situ* virus detection on the field to avoid further viral dissemination or breakouts (6). This emergency response program should be implemented to safeguard biodiversity following the One Health principles (23) in accordance with the United Nations Decade of Ocean Science for Sustainable Development (e.g., Sustainable Development Goals, SDG 14: life below water) and the Kumming-Montreal Global Biodiversity framework.

Low pathogenic avian influenza has previously been documented in seabirds in the Galápagos Islands, while highly pathogenic avian influenza (HPAI, specifically H5N1) has not yet been identified. The HPAI H5N1 2.3.4.4b clade was identified as the cause of the massive poultry mortality in continental Ecuador (21), located 1,000 km from the Galápagos Islands) and detection of CDV in domestic and feral dogs and subsequent isolation from dead Galápagos sea lion pups establishes a precedent for horizontal transmission of highly infectious pathogens. Throughout the northern and southern hemispheres, there are profound disparities in case reporting and surveillance efforts. In the Galápagos Islands, heightening public awareness through education campaigns and contact with veterinarians is recommended. Any birds that present neurologic signs, including ataxia, torticollis, or hemiparesis, should be reported to the Galápagos National Park and Ecuador's Ministry of Environment, Water and Ecological Transition. Appropriate personal protective equipment and rigorous biosecurity measures should be instituted with suspect cases.

While the H5N1 viruses continue to spread globally in record numbers and diversity of affected species, the source of infection among marine mammal species such as seals and dolphins is still poorly understood (44). Increasing surveillance for infection in marine mammals is important in assessing the risk of transmission. As these viruses continue to evolve, employing One Health measures would be prudent to limit exposures and prevent their spread including enhanced surveillance, rapid diagnostics and collaboration between animal and human sectors. Based on prior knowledge, the introduction of H5N1 into the local Galápagos sea lion and fur seal populations could have catastrophic outcomes.
