# Supplementary material for: Endangered Galápagos sea lions and fur seals under the siege of lethal avian flu: a cautionary note on emerging infectious viruses in endemic pinnipeds of the Galápagos Islands
Source: Front Vet Sci. 2024 Sep 20;11:1457035. doi: 10.3389/fvets.2024.1457035 (PMC11449844; doi:10.3389/fvets.2024.1457035)
Supplement: Supplementary file 1 [file Table_1.docx]

**Supplementary Information**

**Endangered Galápagos sea lions and fur seals under the siege of lethal avian flu: A cautionary note on emerging infectious viruses in endemic pinnipeds of the Galápagos Islands**

Juan José Alava^1,2,3*^, Ana Tirapé^4^, Judith Denkinger^5,6^, Paola Calle^4^, Patricia Rosero R.^7^,

Sandie Salazar^5^, Patricia A. Fair^8^, Stephen Raverty^1,9^

^1^Ocean Pollution Research Unit, Institute for the Oceans and Fisheries, University of British Columbia, Vancouver, BC V6T 1Z4, Canada

^2^School of Resource and Environmental Management, Simon Fraser University, Burnaby, BC V5A 1S6, Canada

^3^Fundación Ecuatoriana para El Estudio de Mamíferos Marinos (FEMM), Guayaquil, Ecuador

^4^ Escuela Superior Politécnica del Litoral, ESPOL, Facultad de Ciencias de la Vida, ESPOL Polytechnic University, Guayaquil, Ecuador,

^5^Universidad San Francisco de Quito, Galápagos Science Center, Campus Cumbaya, Quito, Ecuador

^6^ Ocean Museum, Stralsund, Germany

^7^Escuela de Ciencias Ambientales, Universidad Espíritu Santo, Samborondón, Ecuador.

^8^Department of Public Health Sciences, Medical University of South Carolina, Charleston, South Carolina 29425, United States

^9^Animal Health Centre, British Columbia Ministry of Agriculture, Food and Fisheries, Abbotsford, British Columbia, Canada.

**Correspondence**:

Corresponding Author

Dr. Juan José Alava

E-mail: [j.alava@oceans.ubc.ca](mailto:j.alava@oceans.ubc.ca)

**Table S1**. Timeline records of HPAI A(H5N1) epizootic events reported in marine mammal species from South America in chronological order during the period February 2021 to July 2024.

| **Date** | **Outbreak description** | **References** |
| --- | --- | --- |
| February 2021 | First reported case of HPAI A(H5N1) in wild birds in South America. Suspected transmission to marine mammals under investigation. | (1) |
| November 2022 | An unusual mortality event among sea lions in Peru is reported. Preliminary investigations suggest a potential link to HPAI A(H5N1) infection. | (2,3) |
| December 2022 | Detection of HPAI A(H5N1) in marine mammal carcasses in Chile, including sea lions and fur seals. A regional alert for further investigation is issued. | (4) |
| January 2023 | Significant die-off of marine mammals observed along the coast of Chile and Peru. Genetic sequencing confirms HPAI A(H5N1) in sea lions and fur seals. | (5) |
| April 2023 | Identification of migratory bird patterns showing potential links to HPAI A(H5N1) spread affecting marine mammal populations in South America. | (6) |
| August 2023 | First detection in Argentina (in Rio Grande, Tierra del Fuego Province, Argentina). | (7) |
| September 2023 | Highly pathogenic avian influenza H5N1 virus infections in pinnipeds and seabirds in Uruguay as a paradigm shift to virus transmission in South America. | (8) |
| October 2023 | Brazil reports additional cases of HPAI A(H5N1) in marine mammals, including a notable outbreak among sea lions in the southern part of the country. | (9) |
| October and November 2023 | Report of a massive mortality of southern elephant seals infected with H5N1 avian influenza in Península Valdés, Argentina | (10) |
| December 2023 | Report of the first highly pathogenic avian influenza (H5N1) outbreak in several pinniped species along the southern Brazilian coast | (11) |
| June 2024 | Publication of a comprehensive study on HPAI A(H5N1) impacts on marine mammals, including detailed maps and timelines, highlighting the importance of continued surveillance and research. | (6) |

**References**

1. Heckel G, Schramm Y. Ecology and Conservation of Pinnipeds in Latin America. (Switzerland-11: Springer International Publishing), (2021) 938. doi: 10.1007/978-3-030-63177-2
2. Pan American Health Organization. *Epidemiological Update Outbreaks of Avian Influenza Caused by Influenza A(H5N1) in the Region of the Americas* (2023).
3. Leguia M, Garcia-Glaessner A, Muñoz-Saavedra B, Juarez D, Barrera P, Calvo-Mac C, Jara J, Silva W, Ploog K, Amaro, Lady, et al. Highly pathogenic avian influenza A (H5N1) in marine mammals and seabirds in Peru. *Nat Commun* (2023) 14:5489. doi: 10.1038/s41467-023-41182-0
4. Ulloa M, Fernández A, Ariyama N, Colom-Rivero A, Rivera C, Nuñez P, Sanhueza P, Johow M, Araya H, Torres JC, et al. Mass mortality event in South American sea lions (*Otaria flavescens)* correlated to highly pathogenic avian influenza (HPAI) H5N1 outbreak in Chile. *Vet Q* (2023) 43:1–10. doi: 10.1080/01652176.2023.2265173
5. Gamarra-Toledo V, Plaza PI, Gutiérrez R, Inga-Diaz G, Saravia-Guevara P, Pereyra-Meza O, Coronado-Flores E, Calderón-Cerrón A, Quiroz-Jiménez G, Martinez P, et al. Mass Mortality of Sea Lions Caused by Highly Pathogenic Avian Influenza A(H5N1) Virus. *Emerg Infect Dis* (2023) 29(12):2553–2556. <https://doi.org/10.3201/eid2912.230192>.
6. Plaza P, Gamarra-Toledo V, Rodríguez Euguí J, Rosciano N, Lambertucci S. Pacific and Atlantic sea lion mortality caused by highly pathogenic Avian Influenza A(H5N1) in South America. *Travel Med Infect Dis* (2024) 59(102712)1-4 <https://doi.org/10.1016/j.tmaid.2024.102712>
7. Rimondi A, Vanstreels RET, Olivera V, Donini A, Lauriente MM, Uhart MM. Early Release - Highly Pathogenic Avian Influenza A(H5N1) Viruses from Multispecies Outbreak, Argentina, August 2023. Emerg Infect Dis (2024) 30(4): 812–814. [https://doi.org/10.320 1/eid3004.231725](https://doi.org/10.320%201/eid3004.231725)
8. Tomas G, Marandino A, Panzera Y, Rodríguez S, Wallau G da L, Dezordi F, Pérez R, Bassetti L, Negro R, Williman, J. et al. Highly pathogenic avian influenza H5N1 virus infections in pinnipeds and seabirds in Uruguay: a paradigm shift to virus transmission in South America. *Virus Evol* (2024) 10(1) veae031:1-8 <https://doi.org/10.1093/ve/veae031>
9. Araújo AC, Cho AY, Silva LMN, Correa TC, Souza GC, Albuquerque AS, Macagnan E, Kolesnikvoas CK, Meurer R, Vieira JV, et al. Mortality in Sea Lions is associated with the introduction of the H5N1 clade 2.3. 4.4 b virus in Brazil, October 2023: Whole genome sequencing and phylogenetic analysis. (2024). *BMC Vet Res* 20(285):1-8 <https://doi.org/10.1186/s12917-024-04137-1>
10. Campagna C, Uhart M, Falabella V, Campagna J, Zavattieri V, Vanstreels RET, Lewis MN. Catastrophic mortality of southern elephant seals caused by H5N1 avian influenza. *Mar Mammal Sci* (2024) 40:322–325. doi: 10.1111/mms.13101
11. de Lima RC, Estima SC, Tavares M, Canabarro PL, Botta S, Dias LA, da Silva AP, de Amorim DB, de Oliveira LR. Impacts and lessons learned from the first highly pathogenic avian influenza (H5N1) outbreak in South American pinnipeds along the southern Brazilian coast. *Mar Mammal Sci* (2024) e13163:1-8 <https://doi.org/10.1111/mms.13163>
